# Association between Cardiorespiratory Fitness and Hypertensive Disorders of Pregnancy: A Systematic Review and Meta-Analysis

**DOI:** 10.3390/jcm11154364

**Published:** 2022-07-27

**Authors:** Farah Al-Huda, Gabriel D. Shapiro, Margie H. Davenport, Mariane Bertagnolli, Natalie Dayan

**Affiliations:** 1 Division of Experimental Medicine, Faculty of Medicine and Health Sciences, McGill University Health Centre, Montreal, QC H4A 3J1, Canada; farah.al-huda@mail.mcgill.ca; 2 Department of Epidemiology, Biostatistics and Occupational Health, Faculty of Medicine and Health Sciences, McGill University, Montreal, QC H3A 1G1, Canada; gabriel.shapiro@rimuhc.ca; 3 Program for Pregnancy and Postpartum Health, Faculty of Kinesiology, Sport and Recreation, University of Alberta, Edmonton, AB T6G 2H9, Canada; mdavenpo@ualberta.ca; 4 School of Physical and Occupational Therapy, Faculty of Medicine and Health Sciences, McGill University, Montreal, QC H3G 1Y5, Canada; mariane.bertagnolli@mcgill.ca; 5 Research Institute, McGill University Health Centre, Montreal, QC H4A 3S5, Canada

**Keywords:** cardiorespiratory fitness, hypertensive disorders of pregnancy, gestational hypertension, preeclampsia, pregnancy, physical activity, oxygen consumption, exercise testing

## Abstract

Hypertensive disorders of pregnancy (HDP) are associated with future cardiovascular disease (CVD), which may be mediated by diminished cardiorespiratory fitness (CRF). In this systematic review and meta-analysis, we summarize evidence linking CRF with HDP before, during, and after pregnancy. We searched relevant databases to identify observational or randomized studies that measured CRF (VO_2_ max or peak, VO_2_ at anaerobic threshold, or work rate at peak VO_2_) in women with and without HDP. We pooled results using random effects models. Fourteen studies (n = 2406 women) reporting on CRF before, during, and after pregnancy were included. Before pregnancy, women who developed HDP had lower CRF (e.g., VO_2_max < 37 vs. ≥37 mL O_2_/min) than those without HDP (two studies, 811 women). VO_2_max at 14–18 weeks of pregnancy was marginally lower among women who developed preeclampsia vs. normotensive women (three studies, 275 women; mean difference 0.43 mL/kg/min [95% CI 0.97, 0.10]). Postpartum, there was a trend towards lower VO_2_peak in women with previous preeclampsia (three studies, 208 women; 0.26 mL/kg/min [−0.54, 0.02]). While exploratory, our findings raise the possibility that CRF can identify women at risk for HDP, and furthermore, that HDP confers a hit to a woman’s cardiorespiratory reserve.

## 1. Introduction

Hypertensive disorders of pregnancy (HDP), including gestational hypertension (GH) and preeclampsia (PE), are leading causes of maternal and neonatal morbidity and mortality [1,2]. Beyond the immediate implications for mother and child, numerous studies have demonstrated an association between HDP and future cardiovascular disease (CVD) [3,4,5,6]. However, whether HDP contributes causally to CVD, merely highlights pre-existing CVD risk, or both [7] and what specific mechanisms interact to lead to HDP and future CVD [5] are still uncertain.

Cardiorespiratory fitness (CRF) reflects the combined functional capacity of the cardiovascular and respiratory systems to provide and maintain an adequate oxygen supply to working skeletal muscles over prolonged periods of physical activity [8,9]. Pregnancy alters both the cardiovascular and respiratory systems during rest and exercise, acting as a natural cardiorespiratory stress test [10]. A recent meta-analysis in normotensive women showed that prenatal exercise interventions help improve maternal CRF, indicating that it is an actionable and modifiable metric that has the potential to improve cardiovascular health during and after pregnancy [11].

Focusing on peripartum CRF offers a dynamic and mechanistic approach to understanding the interplay between potential risk factors for HDP and their roles in the trajectory towards CVD [12,13]. However, it is not known whether women who develop HDP have reduced CRF, or if HDP accelerates CRF decline after pregnancy. We therefore conducted this systematic review to summarize the literature on CRF before, during, and after HDP, compared with pregnant women without HDP, with CRF assessed using maximal or peak oxygen consumption (VO_2_), VO_2_ at anaerobic threshold (VO_2_AT), and work rate or test distance or duration.

## 2. Materials and Methods

### 2.1. Study Eligibility and Inclusion Criteria

The Preferred Reporting Items for Systematic Reviews and Meta-Analyses (PRISMA) guidelines were used to conduct this systematic review and meta-analysis based on an a priori protocol [14,15]. The protocol was registered and can be accessed at the International Prospective Register of Systematic Reviews (PROSPERO; Registration No. CRD42019126663). 

We included observational (case–control, prospective and retrospective cohort, and cross-sectional) and interventional (RCT or non-randomized intervention) studies, with no date or language restrictions. Eligible studies were performed in women of any age and parity. Conference abstracts were included only if a corresponding article published in a peer-reviewed journal was not found. Animal studies, case reports and series, comments and editorial letters, and reviews were excluded, as were study protocols. The exposure of interest was HDP of any severity (including PE and gestational hypertension), which needed to be clearly defined either via direct clinical assessment, medical chart review, or by self-report. Studies were only included if there was a control group of unexposed women without HDP, thereby enabling between-group comparisons of outcomes. The outcome in all eligible studies was CRF, measured using any type of cardiopulmonary exercise test or aerobic fitness field test. CRF measures were reported as VO_2_max (mL/kg/min) or VO_2_peak (mL/kg/min), predicted or estimated VO_2_max (mL/kg/min), VO_2_AT (mL/kg/min), and work rate (watts) or aerobic test distance (metres) or duration (seconds). We considered all studies with a measure of HDP and of CRF, irrespective of the timing of these measurements or the presence of other gestational disorders. We reported and analyzed antepartum and postpartum measurements separately. 

### 2.2. Data Sources and Search Strategy

An initial MEDLINE strategy was developed by a research librarian (T.L.) based on a scoping search to identify relevant search terms and was then modified with input from the research team. The final MEDLINE strategy was adapted for other databases, with vocabulary and syntax tailored to enable optimal digital searches up to July 2018 of the following databases: MEDLINE (via Ovid and PubMed), EMBASE, Cochrane Library (CENTRAL and CDSR), and Scopus. Search strategies complied with the Institute of Medicine standards [16] and were not limited by language restrictions. The reference lists of included articles and relevant systematic reviews were checked manually for potentially relevant articles. ClinicalTrials.gov and WHO’s International Clinical Trials Registry Platform (ICTRP) Search Portal were used to identify clinical trials recently completed, and associated publications were then retrieved. A final MEDLINE search was performed to identify additional references published through 14 January 2021 (L.H.). Search terms and results are shown in Appendix A.

### 2.3. Study Selection 

Two reviewers (F.A. and G.D.S.) independently selected studies using the specific eligibility criteria. The first screening was based on titles and abstracts of identified publications. All studies identified by at least one reviewer as potentially relevant were retrieved for full-text evaluation. Both F.A. and G.D.S. independently evaluated full-text references, and reasons for exclusion were recorded. Disagreements were resolved by a third reviewer (N.D.). If studies were found using the same study population, the most recent or most complete publication was selected [17,18,19], unless both publications contained unique, potentially relevant data, in which case both were included but counted as one study in the flow diagram. Citations of relevant systematic reviews and meta-analyses were searched manually (F.A. and G.D.S.), and eligible full texts were retrieved. Authors were contacted when studies reported using cardiopulmonary exercise testing but did not report CRF. For example, studies reporting other cardiorespiratory health measures such as blood pressure and heart rate in response to exercise were excluded if no other CRF values were recorded. References excluded with reasons can be found in Appendix B. 

### 2.4. Data Extraction 

#### 2.4.1. Data Collection Process

Data were extracted from full-text articles using a data extraction spreadsheet in Microsoft Excel by two independent reviewers (F.A. and G.D.S.), with disagreements resolved through discussion and consultation (N.D. and M.H.D.). For the meta-analysis, data were entered into ReviewManager v5.3 (Cochrane Collaboration, Copenhagen, Denmark) by one reviewer and verified by the other (F.A. and G.D.S., respectively). 

#### 2.4.2. Data Retrieved 

Study characteristics extracted included study design, year and country of publication, definition and subtype of HDP (i.e., GH or PE), method of CRF assessment, and type of exercise intervention (if applicable). From each included study, we extracted sample characteristics including number of participants in each exposure group (GH/PE vs. no GH/PE), age, ethnicity, parity, length of time between pregnancy and CRF measurement, whether participants were pregnant or postpartum, history of underlying conditions, CRF measurements, and other obstetric outcomes as available (e.g., gestational age at delivery, Caesarean delivery). 

#### 2.4.3. Assessment of Risk of Bias 

Reviewers (F.A. and G.D.S.) independently assessed the quality of each included study with the resolution of disagreements through discussion or involvement of N.D. Risk of bias was assessed using the Joanna Briggs Institute Critical Appraisal Checklist for Cohort and Analytical Cross Sectional Studies [20], which was used for intervention studies as well, as only pre-intervention data were included. All publications meeting inclusion criteria were included regardless of quality. Given the small number of studies obtained for subgroup analysis and meta-regression, it was not possible to examine the impact of study quality or publication bias on pooled outcomes.

### 2.5. Data Analysis 

Statistical analyses were conducted using ReviewManager. Effect measures were reported as mean differences and standard deviations (SD) in CRF between groups. For studies using different exercise or field test modes to estimate CRF, standardised mean differences (SMD) were calculated instead. If studies reported standard error (SE), SD values were calculated from the SE by multiplying by the square root of the sample size [21]. If results were reported as absolute CRF, authors were contacted to obtain CRF relative to weight. 

Meta-analyses were conducted using inverse variance random-effects models. If there were at least 2 studies using the same type of variable for CRF, results were pooled, with a *p*-value < 0.05 considered statistically significant. The I-squared statistic was used to evaluate heterogeneity, with results pooled only for studies with I^2^ < 80%. A priori subgroup analyses were carried out for type of CRF measurement (VO_2_peak, estimated VO_2_max, VO_2_AT, work rate, test distance, or test time) and timing of CRF measurement (before, during, or after pregnancy). If data were not suitable for meta-analysis or relevant data were missing or unclear, authors were contacted to obtain additional information. Data were qualitatively synthesized if authors were unable to provide additional numerical data.

## 3. Results

### 3.1. Literature Search

**Figure 1:**
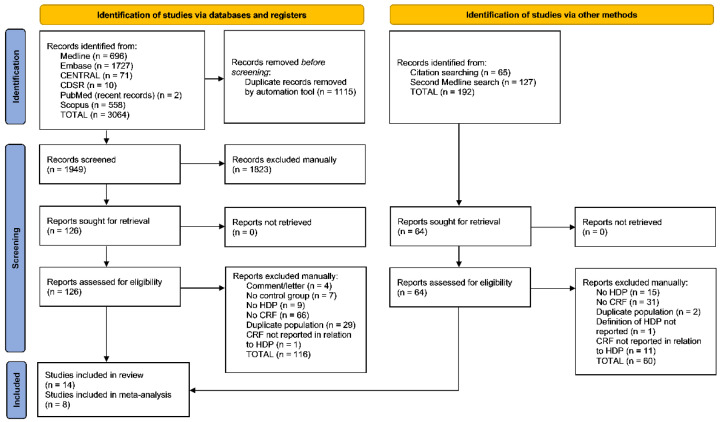
PRISMA flow diagram.

The initial literature search yielded 1949 records for title and abstract screening after removal of duplicate results, of which 126 records were retrieved and assessed for eligibility. A total of 116 reports were excluded for reasons including no measure of any HDP or CRF, no normotensive comparison group, CRF not reported in relation to HDP, duplicate study population, or inappropriate study design (Figure 1). Ten studies were included in the review [17,18,19,22,23,24,25,26,27,28] and five in the meta-analysis [17,19,22,23,28]. Manual citation searching of included articles and relevant systematic reviews yielded 65 additional references, of which 3 were included in our systematic review [29,30,31] (2 in the meta-analysis [29,31]). Finally, the second Medline search conducted on 14 January 2021 yielded 127 additional records for title and abstract screening, among which 13 additional eligible reports were assessed, and 1 [32] was included in the meta-analysis. Authors were contacted to request study data for nine studies for which data relevant to our review were potentially available but not reported [18,19,23,25,27,28,29,30,32]; authors of seven studies responded and provided relevant data [19,23,25,28,29,30,32]. A total of 14 studies met final inclusion criteria including 2406 women: 560 with HDP GH/PE and 1846 without GH/PE.

### 3.2. Study Characteristics 

Eight cohort studies [18,22,25,26,27,28,31,32], four RCTs [19,23,29,30], one cross-sectional study [24], and one non-randomized intervention study [17] were included (Table 1). The number of included participants in each study ranged from 26 to 768, while the number of participants with GH/PE ranged from 3 to 139. Estimated VO_2_max was measured in four studies [18,19,28,29], VO_2_peak in three studies [17,22,32] and VO_2_AT in one study [30]. Of those that did not measure VO_2_, two studies used a Balke treadmill test [26,28], one used a cycle ergometer test (pulse-rate-controlled submaximal 6 min exercise test with target heart rate ≥ 140 bpm) [31], and three used a walk or run test (6 min walk test, 4-m walk test, 2-mile walk or run test) [23,24,25]. CRF was examined before [18,27], during [19,23,24,28,29,30,31], and after [17,22,23,25,26,32] pregnancy in two, seven, and six studies, respectively (one study examined CRF both during pregnancy and postpartum [23]). Among studies that examined CRF during pregnancy, CRF was measured before diagnosis of HDP in four studies [19,23,28,29] (range 12 to 18 weeks gestation) and after diagnosis of HDP in one study [31] (35.5 to 37.7 weeks gestation), while one study included serial measurements of CRF [30] (14 and 28 weeks gestation). Eight studies contained sufficient data to be included in the meta-analysis [17,19,22,23,28,29,31,32]; results from the remaining six studies were reported qualitatively [18,24,25,26,27,30]. Of the four RCTs, two [29,30] reported clinical trial registration.

### 3.3. Study Quality

Study quality ratings are shown in Table 2. Appropriate conduct of analysis and follow-up was documented in nearly all studies. Exposure measurement was considered valid and reliable in 12 of 14 studies. One study was presented as an abstract, and women with previous severe PE were compared to controls, but assessment of PE was not well-described [22]. The second study reported mean watts produced but did not report VO_2_ [23]. The outcome was measured in a valid and reliable way in 8 [17,19,22,23,28,29,30,32] of 14 studies. Unreliable outcome measures for the purposes of our review included self-reported HDP [27], assessment of HDP without reporting diagnostic criteria [18], and measurement of work rate [31], distance travelled [24,25], or exercise test duration [26] without presentation of sufficient data to calculate VO_2_.

Confounding factors such as maternal ethnicity, smoking, education, and income were identified in 13 of 14 studies, and strategies to deal with confounding factors were stated in 9 of these studies. However, none of the studies included adjusted analyses of the association between HDP and CRF.

### 3.4. Study Description

#### 3.4.1. CRF before Pregnancy and Subsequent HDP

Two studies [18,27] examined CRF before pregnancy in relation to future GH and/or PE. Morris et al. [18] compared women with VO_2_max < 37 vs. ≥37 mL O_2_/min and found that pre-pregnancy CRF was low in 100% of women with GH or PE (n = 10) compared to 57.6% (19/33) of women without GH or PE (*p* = 0.01). Lane-Cordova et al. [27] divided study participants into three fitness tertiles based on performance on a treadmill test and found that GH was diagnosed in 21%, 19%, and 12% of women in the lowest, middle, and highest fitness tertiles, respectively (*p* = 0.03). However, the study did not report rates of PE.

#### 3.4.2. CRF during Pregnancy and Subsequent GH or PE

Seven studies [19,23,24,28,29,30,31] examined CRF during pregnancy. Bisson et al. [30] found a mean VO_2_AT of 15.0 and 15.7 mL/kg/min among women who developed GH and normotensive women, respectively, at 14 weeks gestation, with a mean VO_2_AT of 14.9 and 15.0 mL/kg/min in the two groups, respectively, at 28 weeks gestation. Da Silva et al. [24] found women who had been diagnosed with PE completed a shorter distance on the 6 min walk test compared to normotensive women (421 m vs. 497 m, *p* = 0.001, median gestational age at measurement 37 weeks in both groups, interquartile range 34–38 among women with PE, 33–38 for normotensive women). 

**Figure 2:**
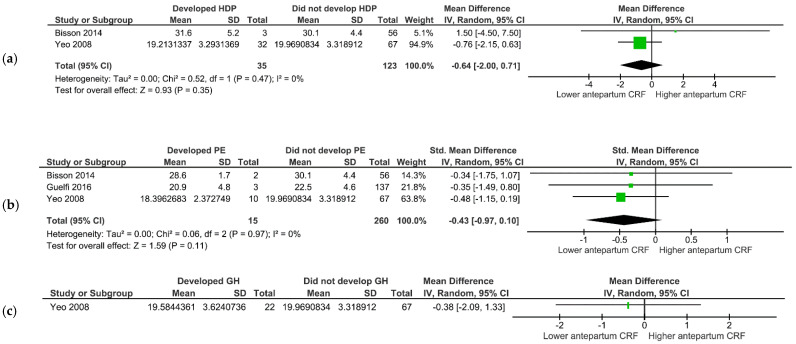
Mean differences in VO_2_max during pregnancy and subsequent development of preeclampsia and/or gestational hypertension: (**a**) Weight-adjusted VO_2_max (in mL/kg/min) and preeclampsia or gestational hypertension [19,28]; (**b**) Weight-adjusted VO_2_max (in mL/kg/min) and preeclampsia [19,28,29]; (**c**) Weight-adjusted VO_2_max (in mL/kg/min) and gestational hypertension [19].

Estimated VO_2_max measured during pregnancy did not significantly differ between women who developed GH and/or PE combined and those who did not (two studies [19,28], 158 women, gestational age at measurement 16–18 weeks; −0.64 mL/kg/min [95% CI −2.00, 0.71]; *p* = 0.35) (Figure 2a). Similarly, no difference in VO_2_max was found between women who developed GH only vs. non-HDP women (one study [19], 146 women, gestational age at measurement 18 weeks; −0.38 mL/kg/min [95% CI −2.09, 1.33]; *p* = 0.66) (Figure 2c). However, a trend toward lower VO_2_max was observed for women who developed PE only vs. non-HDP women (three studies [19,28,29], 275 women, gestational age at measurement 14–18 weeks; −0.43 mL/kg/min [−0.97, 0.10]; *p* = 0.11) (Figure 2b). 

The mean work rate did not significantly differ between groups (two studies [23,31], 88 women; −0.08 watts [−1.01, 0.85]; *p* = 0.17; Figure S2), nor did lactate threshold (one study [30], 47 women; −0.70 mL/kg/min [−2.25, 0.85] at 14 weeks gestation; −0.10 mL/kg/min [−1.49, 1.29] at 28 weeks gestation).

Three studies measured absolute VO_2_max during pregnancy without adjustment for weight, rendering values challenging to interpret. Absolute VO_2_max was significantly higher in women with GH or PE vs. those without (two studies [19,28], 163 women, gestational age at measurement 16–18 weeks; 0.19 L/min [0.03, 0.35]; *p* = 0.02) and in women with GH only vs. those without (one study [19], 94 women, gestational age at measurement 18 weeks; 0.54 L/min [0.08, 1.01]; *p* = 0.02), while it was marginally higher in women with PE only vs. those without (three studies [19,28,29], 277 women, gestational age at measurement 14–18 weeks; 0.96 mL/kg/min [−0.23, 2.14]; *p* = 0.11) (Figure S1). 

#### 3.4.3. HDP and Postpartum CRF

**Figure 3:**
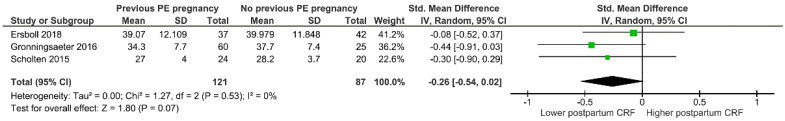
Mean differences in VO_2_peak (in mL/kg/min) in postpartum women with and without previous preeclampsia [17,22,32].

CRF was examined after pregnancy in six studies [17,22,23,25,26,32]. Duration since delivery at the time of CRF measurement ranged from 6 weeks to 20 years. There was a trend towards lower VO_2_peak in women with prior PE compared with controls (three studies [17,22,32], 208 women; −0.26 mL/kg/min [−0.54, 0.02], *p* = 0.07; Figure 3). Cottrill et al. [26] found that women with previous GH and/or PE had a lower mean duration of exercise on a modified Balke treadmill test compared to women with previous non-HDP pregnancy (115 women; 660 vs. 738 s; SMD −0.42 [−0.80, 0.05]; *p* = 0.03; measurement of CRF 4–6 years following pregnancy). Harville et al. [25] found that women with GH and/or PE took longer to complete a 4-m walk test compared to women with no previous GH or PE (1329 women; 4.11 vs. 3.95 s; 0.18 [−0.00, 0.36]; *p* = 0.05), with similar distances completed on a 6 min walk test (1246 women; 409.7 vs. 413.3 m; −0.05 [−0.24, 0.15]; *p* = 0.61) (median age at last pregnancy 28.0, median age at interview 48.4).

## 4. Discussion

In this systematic review and meta-analysis of 14 studies, we identified a pattern of lower preconception CRF in women who were subsequently diagnosed with GH and/or PE. While no significant differences were noted in weight-adjusted CRF during pregnancy, results suggest that women who developed PE may have lower CRF earlier in pregnancy, as well as months to years after delivery. 

It is widely known that higher CRF improves cardiovascular health and lowers cardiovascular mortality [33,34]. Previous studies have shown HDP, especially preeclampsia, to be a risk factor for later CVD [3,4], with a two-fold increased risk of ischemic heart disease 10 to 15 years following the pregnancy [5]. Our findings suggest that reduced CRF may be one pathway connecting HDP to future adverse cardiovascular outcomes. Specifically, lower postpartum CRF in HDP-affected women suggests a possible “hit” to cardiorespiratory reserve brought about by HDP—one that is potentially actionable.

Our study adds to the large body of literature suggesting that pregnancy is a “stress test” identifying women at risk for future cardiovascular risk factors and overt CVD [35]. Our analyses suggest reduced cardiorespiratory capacity both in women who went on to develop GH or PE and in those with previous HDP. Prior systematic reviews have demonstrated that engaging in physical activity during pregnancy reduces the odds of developing HDP by ~40% [36]. That this observed benefit seems mediated by improved CRF is perhaps not surprising but highlights the importance of offsetting the complex cardiovascular adaptations of both normotensive and hypertensive pregnancy with exercise. It is therefore likely that CRF is a metric that can be used both to identify higher-risk individuals and also to personalize safe and targeted exercise programs during and after pregnancy.

Our study highlights the need for the standardization of exercise tests used in research on pregnant and postpartum women in order to facilitate meaningful comparison of results between studies. Our findings also have implications for clinical practice. The American Heart Association has recommended that CRF be used as a risk marker for morbidity and mortality in the general population [12], and our review suggests that CRF may be particularly valuable as a clinical vital sign in the peripartum period and during reproductive years. CRF may also prove useful in clinical prediction of maternal morbidity, as adverse cardiovascular outcomes make up a substantial proportion of severe maternal morbidity [37,38]. Finally, our findings suggest that dedicated postpartum cardiovascular rehabilitation after HDP may be worthwhile, using a patient-centred approach sensitive to the needs of new mothers.

In light of evidence for the benefits of physical activity during pregnancy [39], the appropriateness of physical activity in women with established HDP is an area under study. Current GH is a relative contraindication to physical activity, whereas PE is an absolute contraindication [40,41]. Our review was not designed to assess whether current guideline recommendations regarding physical activity for women with HDP are appropriate, and further studies to elucidate the safe CRF threshold customization of physical activity regimens for women with HDP under the guidance of obstetrician-gynecologists or other obstetric care providers, such as kinesiologists, are essential [42].

Our review is subject to several limitations. While the included studies showed high quality ratings with regards to selection of participants and measurement of exposures and covariates, many studies used CRF measurements that did not allow calculation of VO_2_. Accordingly, our ability to combine results from different studies in meta-analysis was limited by the variety of CRF measures employed, as well as by the heterogeneity of study designs. Standardization of CRF measures in perinatal research, including adjustment for BMI, will be essential to understanding the role of CRF in normotensive and hypertensive pregnancy, including its lasting effects in the postpartum period. In addition, most of the included studies did not explicitly aim to measure HDP in relation to CRF and examined HDP as a secondary outcome. Furthermore, the majority of studies examining CRF during pregnancy measured CRF before HDP diagnosis, limiting our ability to draw conclusions about the effects of HDP on CRF in a current pregnancy. Finally, while preeclampsia is understood as a heterogeneous disorder with different phenotypes during pregnancy (i.e., early-onset vs. late-onset) [43], the majority of studies included in this review did not report on the timing or severity of HDP. In order to plan tailored postpartum rehabilitation programs, further studies should evaluate CRF according to the timing and severity of HDP.

HDP often occurs in women with existing comorbidities, such as pregestational diabetes and obesity, which may impact cardiovascular fitness. Women with pre-pregnancy obesity are particularly at elevated risk for PE and CVD and also tend to have reduced CRF. While our analyses focused on weight-adjusted CRF, several of the studies included in our review examined VO_2_ without adjustment for maternal weight, which tends to be higher in women with HDP. Additional studies using weight-adjusted VO_2_ values before, during, and after HDP are needed to analyze CRF changes with respect to HDP and to shed light on the interplay between HDP and CVD risk factors.

Our analyses may also be subject to confounding by age, which is known to be linked with CRF decline. However, the relatively narrow age range of child-bearing women likely minimizes the importance of this bias. Finally, we were not able to address persistence and duration of hypertension after delivery. However, PE generally normalizes within one to two weeks postpartum in 70% of individuals [44], whereas the earliest postpartum CRF measurements in the included studies were at six weeks.

Despite these limitations, our study was focused on CRF as an actionable risk marker rather than an independent causal factor. Furthermore, our aim was to identify knowledge gaps to guide future research in the prospective evaluation of CRF. Eventually, studies evaluating CRF-guided rehabilitation programs at key periods in women’s reproductive trajectory will be needed to contribute evidence-based recommendations for optimal cardiovascular health around the time of pregnancy. 

## 5. Conclusions

In summary, in the first systematic review to date on CRF before, during, and after HDP suggested that CRF is a valuable marker of perinatal cardiovascular risk, and that postpartum measurement of CRF may shed light on the cardiovascular sequelae of hypertensive pregnancy. Our findings raise the possibility that HDP may impair cardiorespiratory reserve and suggest that dedicated postpartum cardiovascular rehabilitation programs may be indicated. Additional studies using standardized measures of VO_2_ are needed to quantify the strength and temporality of the association between CRF and HDP.

## Figures and Tables

**Table 1:** Study and participant characteristics.

Reference	Study Design	HDP Diagnosis	CRF Measures	Total N	HDP n	Age (Mean + Standard Deviation) or (Median, Range), Years	Parity	CRF Measurement Timing
Bisson 2014 [28]	Cohort	GH and PE from chart review at 36 weeks GA	Estimated VO_2_max (mL/kg/min) Modified Balke treadmill test	59	3	30 ± 4.5	43% multiparous	16 weeks GA
Gronningsaeter 2016 [22]	Cohort	PE from chart review or self-report	VO_2_peak (mL/kg/min) Treadmill test	85	60	Control: 38 ± 4 PE: 41 ± 4	Not reported	Postpartum
Guelfi 2016 [29]	RCT	PE from chart review at 37–40 weeks GA	Estimated VO_2_max (mL/kg/min) Cycle ergometer test	140	3	Control: 33.8 ± 3.9 Exercise: 33.6 ± 4.1	Control: 18% multiparous Exercise: 32% multiparous	14 weeks GA
Scholten 2015 [17]	Non-randomized intervention	PE from chart review	VO_2_peak (mL/kg/min) Cycle ergometer test	44	24	32 ± 4	Primiparous	7 ± 2 months postpartum
Yeo 2008 [19]	RCT	GH and PE from chart review	Estimated VO_2_max (mL/kg/min) Cornell treadmill test	102	32	72% aged 20–34	Not reported	18 weeks GA
Bisson 2015 [30]	RCT	GH and PE from chart review	VO_2_AT using V-slope method Modified Bruce treadmill peak XT	48	5	Control: 31 ± 4 Exercise: 30.5 ± 3.7	Control: 56% multiparous Exercise: 56% multiparous	14, 28 weeks GA
Cottrill 1980 [26]	Cohort	HDP from clinical measurements	Test duration (s) Modified Balke treadmill test	115	63	HDP: 21 (range 17–24) No HDP: 19 (range 17–25)	Primiparous	Postpartum
da Silva 2010 [24]	Cross-sectional	PE from chart review	Test distance (m) 6 min walk test	74	37	PE: 21 (range 19–26) No PE: 22 (range 18–24)	Primiparous	37 weeks GA
Harville 2018 [25]	Cohort	HDP from self-report	Test distance (m) 6 min walk test Test duration (s) 4-m walk test	761	139	17.7 ± 5.2	87% multiparous	Postpartum
Price 2012 [23]	RCT	GH from clinical measurements	Work Rate (W) in 2-mile walk or run	62	3	Control: 27.6 ± 7.3 Exercise: 30.5 ± 5.0	Control: 0.67 ± 1 Exercise: 0.5 ± 0.7	12–14 weeks GA through 6–8 weeks postpartum
Rauramo 1988 [31]	Cohort	PE from clinical measurements	Work rate (W) Cycle ergometer test	26	13	PE: 27 ± 4 No PE: 26 ± 3	Not reported	35.5–37.7 weeks GA
Lane-Cordova 2018 [27]	Cohort	Self-reported	Test duration (s) Balke treadmill test	768	129	29 ± 1	Nulliparous	Before pregnancy
Morris 2017 [18]	Cohort	Not reported	VO_2_max (mL/min) Cycle ergometer test	43	10	Unfit group (VO_2_max < 37): 31.4 ± 4.5 Fit group (VO_2_max ≥ 37): 31.8 ± 3.1	Nulliparous	Before pregnancy
Ersboll 2018 [32]	Cohort	Previous PE—ICD-10 diagnosis of severe PE (O14.1)	VO_2_peak (mL/kg/min) Cycle ergometer test	79	39	Uncomplicated: 38.8 ± 5.6 PPCM: 38.0 ± 6.9 Severe PE: 39.1 ± 5.3	Not reported	Median months (IQR) Uncomplicated: 101 (25–146) PPCM: 91 (227–137) Severe PE: 95 (26–143)

**Table 2:** Study quality ratings based on Briggs Institute Critical Appraisal Tools.

Cohort and Intervention Studies	Bisson 2014 [28]	Gronningsaeter 2016 [22]	Guelfi 2016 [29]	Scholten 2015 [17]	Yeo 2008 [19]	Bisson 2015 [30]	Cottrill 1980 [26]	Harville 2018 [25]	Price 2012 [23]	Rauramo 1988 [31]	Lane-Cordova 2018 [27]	Morris 2017 [18]	Ersboll 2018 [32]
Were the two groups similar and recruited from the same population?	✓	U	✓	–	✓	✓	✓	✓	✓	U	✓	✓	✓
Were the exposures measured similarly to assign people to both exposed and unexposed groups?	✓	U	✓	✓	✓	✓	✓	✓	✓	✓	✓	✓	✓
Was the exposure measured in a valid and reliable way?	✓	U	✓	✓	✓	✓	✓	✓	U	✓	✓	✓	✓
Were confounding factors identified?	✓	–	✓	✓	✓	✓	✓	✓	✓	✓	✓	✓	✓
Were strategies to deal with confounding factors stated?	✓	–	✓	–	✓	✓	–	✓	–	✓	✓	–	✓
Were the groups/participants free of the outcome at the start of the study (or at the moment of exposure)?	✓	N/A	✓	N/A	✓	✓	N/A	N/A	✓	N/A	U	✓	N/A
Were the outcomes measured in a valid and reliable way?	✓	✓	✓	✓	✓	✓	U	U	✓	U	–	–	✓
Was the follow-up time reported and sufficient to be long enough for outcomes to occur?	✓	U	✓	✓	✓	✓	✓	✓	✓	✓	✓	–	✓
Was follow-up complete, and if not, were the reasons to loss to follow-up described and explored?	✓	✓	✓	✓	✓	✓	–	U	✓	✓	✓	–	✓
Were strategies to address incomplete follow-up utilized?	N/A	N/A	N/A	N/A	N/A	N/A	U	U	N/A	N/A	N/A	–	N/A
Was appropriate statistical analysis used?	✓	✓	✓	✓	✓	✓	✓	✓	✓	✓	✓	✓	✓
**Cross-sectional studies**	da Silva 2010 [24]												
Were the criteria for inclusion in the sample clearly defined?	✓												
Were the study subjects and the setting described in detail?	✓												
Was the exposure measured in a valid and reliable way?	✓												
Were objective, standard criteria used for measurement of the condition?	✓												
Were confounding factors identified?	✓												
Were strategies to deal with confounding factors stated?	✓												
Were the outcomes measured in a valid and reliable way?	U												
Was appropriate statistical analysis used?	✓												

✓, Yes; –, No; U, Unclear; N/A, Not applicable.

## Data Availability

Data available on request.

## Supplementary Materials

The following are available online at https://www.mdpi.com/article/10.3390/jcm11154364/s1. Figure S1: Mean weight-unadjusted differences in VO_2_max during pregnancy and subsequent development of preeclampsia and/or gestational hypertension; Figure S2: Mean difference in work rate (Watts) during pregnancy and development of preeclampsia and/or gestational hypertension.

## Appendix A

**Table A1:** Database search strategy.

**Ovid Medline**		
**#**	**Searches**	**Results**
1	exp Hypertension, Pregnancy-Induced/	33,283
2	Hypertension/	220,828
3	limit 2 to yr = “1970–2004”	121,134
4	pregnancy/	819,642
5	3 and 4	6568
6	Pregnancy Complications, Cardiovascular/	15,517
7	limit 6 to yr = “1970–2004”	9895
8	(eclamp* or preclamp* or preeclamp*).tw,kf.	31,687
9	(EPH adj3 (complex* or gestosis or toxemia* or toxaemia*)).tw,kf.	484
10	(PIH or PPEP).tw,kf.	1881
11	((pregnant or pregnancy or pregnancies or maternal or gestation* or proteinuria or gestosis) adj3 (hypertens* or hyper-tens* or toxemia* or toxaemia*)).tw,kf.	20,294
12	1 or 5 or 7 or 8 or 9 or 10 or 11	61,024
13	exp Exercise/	166,754
14	Exercise Test/	58,836
15	Exercise Tolerance/	10,971
16	exp Oxygen Consumption/	101,596
17	exp Physical Fitness/	26,082
18	((bruce or naughton or ramp) adj2 protocol*).tw,kf.	1516
19	((cardiopulmonary or cardio-pulmonary or cardiorespiratory or cardio-respiratory or physical) adj3 fitness).tw,kf.	13,302
20	((aerobic* or cardiopulmonary or cardio-pulmonary or cardiorespiratory or cardiorespiratory or cycl*-ergomet* or exercise or fitness or rockport or stress or treadmill or walk*) adj3 (capacity or test*)).tw,kf.	73,422
21	GXT.tw,kf.	300
22	LTPA.tw,kf.	693
23	(metabolic-equivalen* or METs).tw,kf.	10,280
24	(oxygen adj2 consum*).tw,kf.	38,160
25	Oxygenat*.tw,kf.	60,929
26	physical-activit*.tw,kf.	92,897
27	(vo2max or vo2-max).tw,kf.	10,131
28	or/13–27	468,670
29	12 and 28	733
30	Animals/ not Humans/	4,436,891
31	(animals or animal or canine* or cat or cats or dog or dogs or feline or hamster* or	2,282,522
	mice or monkey or monkeys or mouse or murine or pig or pigs or piglet* or porcine or primate* or rabbit* or rats or rat or rodent* or sheep or veterinar*).ti,jw.	
32	30 or 31	4,854,610
33	29 not 32	698
34	remove duplicates from 33	696
**PubMed**		
Search	Query	Items found
#21	Search (#19) AND #20	2
#20	Search 2018/07/05[CRDT] OR 2018/07/04[CRDT] OR 2018/07/03[CRDT] OR 2018/07/02[CRDT] OR 2018/07/01[CRDT] OR 2018/06/30[CRDT] OR 2018/06/29[CRDT]	22,890
#19	Search (#17) NOT #18	1552
#18	Search (animals[Title] OR animal[Title] OR canine*[Title] OR cat[Title] OR cats[Title] OR dog[Title] OR dogs[Title] OR feline[Title] OR hamster*[Title] OR mice[Title] OR monkey[Title] OR monkeys[Title] OR mouse[Title] OR murine[Title] OR pig[Title] OR pigs[Title] OR piglet*[Title] OR porcine[Title] OR primate*[Title] OR rabbit*[Title] OR rats[Title] OR rat[Title] OR rodent*[Title] OR sheep[Title] OR veterinar*[Title])	2,054,960
#17	Search (#5) AND #16	1649
#16	Search (((((((((#6) OR #7) OR #8) OR #9) OR #10) OR #11) OR #12) OR #13) OR #14) OR #15	559,635
#15	Search (vo2max[Text Word] OR vo2-max[Text Word])	9783
#14	Search physical-activit*[Text Word]	92,749
#13	Search oxygenat*[Text Word]	72,441
#12	Search oxygen-consum*[Text Word]	113,396
#11	Search (metabolic-equivalen*[Text Word] OR METs[Text Word])	10,309
#10	Search LTPA[Text Word]	691
#9	Search GXT[Text Word]	297
#8	Search ((aerobic*[Text Word] OR cardiopulmonary[Text Word] OR cardiopulmonary[Text Word] OR cardiorespiratory[Text Word] OR cardio- respiratory[Text Word] OR cycl*-ergomet*[Text Word] OR exercise[Text Word] OR fitness[Text Word] OR rockport[Text Word] OR stress[Text Word] OR treadmill[Text Word] OR walk*[Text Word])) AND (capacity[Text Word] OR test[Text Word] OR tests[Text Word] OR testing[Text Word])	304,975
#7	Search ((cardiopulmonary[Text Word] OR cardio-pulmonary[Text Word] OR cardiorespiratory[Text Word] OR cardio-respiratory[Text Word] OR physical[Text Word])) AND fitness[Text Word]	36,809
#6	Search ((bruce[Text Word] OR naughton[Text Word] OR ramp[Text Word]))	1854
	AND protocol*[Text Word]	
#5	Search (((#1) OR #2) OR #3) OR #4	71,017
#4	Search ((pregnant[Text Word] OR pregnancy[Text Word] OR pregnancies[Text Word] OR maternal[Text Word] OR gestation*[Text Word] OR proteinuria[Text Word] OR gestosis[Text Word])) AND (hypertens*[Text Word] OR hypertens*[Text Word] OR toxemia*[Text Word] OR toxaemia*[Text Word])	43,076
#3	Search (PIH[Text Word] OR PPEP[Text Word])	1858
#2	Search (EPH[Text Word]) AND (complex*[Text Word] OR gestosis[Text Word] OR toxemia*[Text Word] OR toxaemia*[Text Word])	820
#1	Search (eclamp*[Text Word] OR preclamp*[Text Word] OR preeclamp*[Text Word])	42,733
**Ovid Embase**		
**#**	**Searches**	**Results**
1	maternal hypertension/	15,415
2	pregnancy toxemia/	3503
3	exp “eclampsia and preeclampsia”/	56,959
4	((pregnant or pregnancy or pregnancies or maternal or gestation* or proteinuria or gestosis) adj3 (hypertens* or hyper-tens* or toxemia* or toxaemia*)).tw,kw.	28,915
5	(eclamp* or preclamp* or preeclamp*).tw,kw.	48,554
6	(EPH adj3 (complex* or gestosis or toxemia* or toxaemia*)).tw,kw.	616
7	(PIH or PPEP).tw,kw.	2730
8	or/1–7	84,051
9	exp exercise/	318,026
10	exp exercise test/	78,173
11	exercise tolerance/	15,747
12	oxygen consumption/	109,537
13	anaerobic threshold/	3516
14	anaerobic capacity/	2084
15	metabolic equivalent/	2376
16	exp physical activity/	368,549
17	fitness/	38,060
18	cardiorespiratory fitness/	2855
19	((bruce or naughton or ramp) adj2 protocol*).tw,kw.	2693
20	((cardiopulmonary or cardio-pulmonary or cardiorespiratory or cardio-respiratory or physical) adj3 fitness).tw,kw.	17,865
21	((aerobic* or cardiopulmonary or cardio-pulmonary or cardiorespiratory or cardiorespiratory or cycl*-ergomet* or exercise or fitness or rockport or stress or treadmill or walk*) adj3 (capacity or test*)).tw,kw.	110,314
22	GXT.tw,kw.	396
23	LTPA.tw,kw.	786
24	(metabolic-equivalen* or METs).tw,kw.	17,265
25	(oxygen adj2 consum*).tw,kw.	53,643
26	oxygenat*.tw,kw.	85,381
27	physical-activit*.tw,kw.	128,981
28	(vo2max or vo2-max).tw,kw.	13,276
29	or/9–28	928,254
30	8 and 29	1843
31	(animal experiment/ or experimental animal/) not human/	2,021,318
32	(animals or animal or canine* or cat or cats or dog or dogs or feline or hamster* or mice or monkey or monkeys or mouse or murine or pig or pigs or piglet* or porcine or primate* or rabbit* or rats or rat or rodent* or sheep or veterinar*).ti,jx.	2,737,925
33	31 or 32	3,745,193
34	30 not 33	1762
35	remove duplicates from 34	1727
**The Cochrane Library**		
ID	SEARCH	HITS
#1	(eclamp* or preclamp* or preeclamp*):ti,ab,kw	2356
#2	(EPH near/3 (complex* or gestosis or toxemia* or toxaemia*)):ti,ab,kw	11
#3	(PIH or PPEP):ti,ab,kw	170
#4	((pregnant or pregnancy or pregnancies or maternal or gestation* or proteinuria or gestosis) near/3 (hypertens* or hyper-tens* or toxemia* or toxaemia*)):ti,ab,kw	1654
#5	[or #1–#4]	3416
#6	((bruce or naughton or ramp) near/2 protocol*):ti,ab,kw	352
#7	((cardiopulmonary or cardio-pulmonary or cardiorespiratory or cardiorespiratory or physical) near/3 fitness):ti,ab,kw	4906
#8	((aerobic* or cardiopulmonary or cardio-pulmonary or cardiorespiratory or cardio-respiratory or cycl*-ergomet* or exercise or fitness or rockport or stress or treadmill or walk*) near/3 (capacity or test*)):ti,ab,kw	23,206
#9	GXT:ti,ab,kw	73
#10	LTPA:ti,ab,kw	50
#11	(metabolic-equivalen* or METs):ti,ab,kw	1609
#12	(oxygen near/2 consum*):ti,ab,kw	9321
#13	oxygenat*:ti,ab,kw	5821
#14	physical-activit*:ti,ab,kw	18,393
#15	(vo2max or vo2-max):ti,ab,kw	2020
#16	[or #6–#15]	52,934
#17	#5 and #16	81
CDSR (to issue 7 of 12, July 2018): 10 CENTRAL (to issue 6 of 12, June 2018): 71		
**Scopus**		
ID	SEARCH	HITS
#1	TITLE-ABS(eclamp* or preclamp* or preeclamp*)	36,278
#2	TITLE-ABS(EPH w/3 (complex* or gestosis or toxemia* or toxaemia*))	618
#3	TITLE-ABS(PIH or PPEP)	2392
#4	TITLE-ABS((pregnant or pregnancy or pregnancies or maternal or gestation* or proteinuria or gestosis) w/3 (hypertens* or hyper-tens* or toxemia* or toxaemia*))	24,068
#5	#1 OR #2 OR #3 OR #4	54,422
#6	TITLE-ABS((bruce or naughton or ramp) w/2 protocol*)	1928
#7	TITLE-ABS((cardiopulmonary or cardio-pulmonary or cardiorespiratory or cardio-respiratory or physical) w/3 fitness)	18,136
#8	TITLE-ABS((aerobic* or cardiopulmonary or cardio-pulmonary or cardiorespiratory or cardio-respiratory or cycl*-ergomet* or exercise or fitness or rockport or stress or treadmill or walk*) w/3 (capacity or test*))	131,605
#9	TITLE-ABS(GXT)	392
#10	TITLE-ABS(LTPA)	749
#11	TITLE-ABS(metabolic-equivalen* or METs)	11,354
#12	TITLE-ABS(oxygen w/2 consum*)	56,425
#13	TITLE-ABS(oxygenat*)	97,183
#14	TITLE-ABS(physical-activit*)	114,817
#15	TITLE-ABS(vo2max or vo2-max)	11,703
#16	#6 OR #7 OR #8 OR #9 OR #10 OR #11 OR #12 OR #13 OR #14 OR #15	406,992
#17	#5 and #16	571
#18	TITLE(animals or animal or canine* or cat or cats or dog or dogs or feline or	2,469,895
	hamster* or mice or monkey or monkeys or mouse or murine or pig or pigs or piglet* or porcine or primate* or rabbit* or rats or rat or rodent* or sheep or veterinar*)	
#19	#17 AND NOT #18	558
**Ovid Medline (12 January 2021)** **Ovid MEDLINE(R) and Epub Ahead of Print, In-Process, and Other Non-Indexed Citations and Daily <1946 to 8 January 2021>**		
**#**	**Searches**	**Results**
1	exp Hypertension, Pregnancy-Induced/	37,290
2	Hypertension/	237,409
3	limit 2 to yr = “1970–2004”	121,132
4	pregnancy/	887,984
5	3 and 4	6568
6	Pregnancy Complications, Cardiovascular/	16,439
7	limit 6 to yr = “1970–2004”	9895
8	(eclamp* or preclamp* or preeclamp*).tw,kf.	37,609
9	(EPH adj3 (complex* or gestosis or toxemia* or toxaemia*)).tw,kf.	494
10	(PIH or PPEP).tw,kf.	2132
11	((pregnant or pregnancy or pregnancies or maternal or gestation* or proteinuria or gestosis) adj3 (hypertens* or hyper-tens* or toxemia* or toxaemia*)).tw,kf.	23,335
12	1 or 5 or 7 or 8 or 9 or 10 or 11	68,956
13	exp Exercise/	202,331
14	Exercise Test/	64,022
15	Exercise Tolerance/	13,058
16	exp Oxygen Consumption/	106,238
17	exp Physical Fitness/	31,176
18	((bruce or naughton or ramp) adj2 protocol*).tw,kf.	1640
19	((cardiopulmonary or cardio-pulmonary or cardiorespiratory or cardio-respiratory or physical) adj3 fitness).tw,kf.	17,027
20	((aerobic* or cardiopulmonary or cardio-pulmonary or cardiorespiratory or cardio- respiratory or cycl*ergomet* or exercise or fitness or rockport or stress or treadmill or walk*) adj3 (capacity or test*)).tw,kf.	86,301
21	GXT.tw,kf.	364
22	LTPA.tw,kf.	865
23	(metabolic-equivalen* or METs).tw,kf.	13,754
24	(oxygen adj2 consum*).tw,kf.	42,190
25	oxygenat*.tw,kf.	72,093
26	physical-activit*.tw,kf.	120,301
27	(vo2max or vo2-max).tw,kf.	11,503
28	or/13–27	548,908
29	12 and 28	860
30	Animals/ not Humans/	4,741,836
31	(animals or animal or canine* or cat or cats or dog or dogs or feline or hamster* or mice or monkey or monkeys or mouse or murine or pig or pigs or piglet* or porcine or primate* or rabbit* or rats or rat or rodent* or sheep or veterinar*).ti,jw.	2,449,689
32	30 or 31	5,225,684
33	29 not 32	820
34	remove duplicates from 33	819
35	(201807* or 201808* or 201809* or 201810* or 201811* or 201812* or 2019* or 2020* or 2021*).dt,ez,ed.	4,305,902
36	34 and 35	144

**Legend for Databases****:** Legends for Medline (Ovid), Embase (Ovid), and CINAHL are available at http://www.muhclibraries.ca/Documents/Database_Legends.pdf (accessed 6 July 2018).

## Appendix B

**Table A2:** References excluded with reasons.

Reasons for Exclusion	References
**Comment/letter**	Amorim MM, Melo AS, Assuncao PL. Comment and reply on-comparison of walking versus stretching exercises to reduce the incidence of preeclampsia: A randomized clinical trial. Hypertens. 2010;29:120–121
	Aparicio VA, Ocon-Hernandez O, Romero L, Soriano-Maldonado A. The role of physical activity on weight gain and hypertensive disorders during pregnancy. Am J Hypertens. 2016;29:e3
	Martin CL, Huber LRB. Physical activity and hypertensive complications during pregnancy: Findings from 2004 to 2006 north carolina pregnancy risk assessment monitoring system. Obstetrical and Gynecological Survey. 2011;66:81–83
	Szabo J, Pal A, Szabo-Nagy A. Preventing toxaemia. Lancet. 2001;357:2140
**No control group**	Aardenburg R, Spaanderman ME, van Eijndhoven HW, de Leeuw PW, Peeters LL. A low plasma volume in formerly preeclamptic women predisposes to the recurrence of hypertensive complications in the next pregnancy. J Soc Gynecol Investig. 2006;13:598–603
	Andolf E, Salminen-Friesendahl C, Thorsell M, Iacobaeus C. Risk factors for cardiovascular disease 11–14 years after severe preeclampsia. Journal of Maternal-Fetal and Neonatal Medicine. 2016;29 (Supplement 1):53–54
	Andolf EG, Iacobaeus C, Nord I. Cardiovascular disease among women with former severe preeclampsia-presence and possible risk factors. Pregnancy Hypertens. 2015;5(1):70
	Hoedjes M, Berks D, Vogel I, Franx A, Oenema A, Duvekot JJ, Habbema JD, Steegers EA, Raat H. Postpartum physical activity after preeclampsia. Pregnancy Hypertens. 2012;2:143–151
	Janmohamed R, Montgomery-Fajic E, Sia W, Germaine D, Wilkie J, Khurana R, Nerenberg KA. Cardiovascular risk reduction and weight management at a hospital-based postpartum preeclampsia clinic. J Obstet Gynaecol Can. 2015;37:330–337
	von Dadelszen P, Magee LA, Devarakonda RM, Hamilton T, Ainsworth LM, Yin R, Norena M, Walley KR, Gruslin A, Moutquin JM, Lee SK, Russell JA. The prediction of adverse maternal outcomes in preeclampsia. Journal of Obstetrics and Gynaecology Canada. 2004;26:871–879
	Zhou MR, Lian MR. Observation of qi-gong treatment in 60 cases of pregnancy-induced hypertension. [chinese]. Zhong xi yi jie he za zhi = Chinese journal of modern developments in traditional medicine/Zhongguo Zhong xi yi jie he yan jiu hui (chou), Zhong yi yan jiu yuan, zhu ban. 1989;9:16–18, 14–185
**No HDP**	Gazioglu K, Kaltreider NL, Rosen M, Yu PN. Pulmonary function during pregnancy in normal women and in patients with cardiopulmonary disease. Thorax 1970;25:445–50.
	Dominguez CG, De Smoler PE, Armas Dominguez J, Karchmer S, Skromne D. [Electrocardiography, stress and the third pregnancy trimester]. Arch Inst Cardiol Mex 1976;46:74–81.
	Hurst AK, Shotan A, Hoffman K, et al. Pharmacokinetic and pharmacodynamic evaluation of atenolol during and after pregnancy. Pharmacotherapy 1998;18:840–6.
	Saldarriaga CI, Franco G, Garzon AM, Garcia I, Mejia N, Restrepo A. [Risk factors for premature coronary disease in women]. Biomedica (Bogota) 2010;30:559–66.
	Chen TH, Hsiao HP, Chiu YW, Shih NH, Chuang HY, Huang CT. Maternal diabetes or hypertension and lifestyle factors may be associated with metabolic syndrome: a population-based study in Taiwan. Kaohsiung J Med Sci 2014;30:86–93.
	Adam C, L’Abbe C, Lachapelle J, et al. Impact of an individualized counselling on physical activity in women with gestational diabetes: Interim analysis of a randomized control trial. Endocrine Reviews Conference: 96th Annual Meeting and Expo of the Endocrine Society, ENDO 2014;35.
	Ueland K, Novy MJ, Metcalfe J. Cardiorespiratory responses to pregnancy and exercise in normal women and patients with heart disease. Am J Obstet Gynecol 1973;115:4–10.
	Bahadoran P, Taebi M, Zolaktaf V, Pouya F. The effect of stretching exercise on changes of blood pressure in nulliparous women. Iran J Nurs Midwifery Res. 2015 March-April; 20(2):205–210.
	Alves E, Henriques A, Correia S, Santos AC, Azevedo A, Barros H. Cardiovascular risk profile of mothers of a Portuguese birth cohort: A survey 4 years after delivery. Prev Med 2013;57:494–9.
	Kordi R, Abolhasani M, Rostami M, Hantoushzadeh S, Mansournia MA, Vasheghani-Farahani F. Comparison between the effect of lumbopelvic belt and home based pelvic stabilizing exercise on pregnant women with pelvic girdle pain; a randomized controlled trial. J Back Musculoskelet Rehabil 2013;26:133–9.
	Haakstad LA, Bo K. Effect of regular exercise on prevention of excessive weight gain in pregnancy: a randomised controlled trial. Eur J Contracept Reprod Health Care 2011;16:116–25.
	Barakat R, Cordero Y, Coteron J, Luaces M, Montejo R. Exercise during pregnancy improves maternal glucose screen at 24–28 weeks: a randomised controlled trial. Br J Sports Med 2012;46:656–61.
	Barakat R, Pelaez M, Montejo R, Luaces M, Zakynthinaki M. Exercise during pregnancy improves maternal health perception: a randomized controlled trial. Am J Obstet Gynecol 2011;204:402.e1–7.
	Hopkins SA, Baldi JC, Cutfield WS, McCowan L, Hofman PL. Exercise training in pregnancy reduces offspring size without changes in maternal insulin sensitivity. J Clin Endocrinol Metab 2010;95:2080-8.
	Perales M, Mateos S, Vargas M, Sanz I, Lucia A, Barakat R. Fetal and maternal heart rate responses to exercise in pregnant women. A randomized Controlled Trial. Arch Med Deporte 2015;32(6):361–367.
	Pelaez M, Gonzalez-Cerron S, Montejo R, Barakat R. Pelvic floor muscle training included in a pregnancy exercise program is effective in primary prevention of urinary incontinence: a randomized controlled trial. Neurourol Urodyn 2014;33:67–71.
	Piravej K, Saksirinukul R. Survey of patterns, attitudes, and the general effects of exercise during pregnancy in 203 Thai pregnant women at King Chulalongkorn Memorial Hospital. J Med Assoc Thai 2001;84 Suppl 1:S276–82.
	Murtezani A, Pacarada M, Ibraimi Z, Nevzati A, Abazi N. The impact of exercise during pregnancy on neonatal outcomes: a randomized controlled trial. J Sports Med Phys Fitness 2014;54:802–8.
	Ussher M, Lewis S, Aveyard P, et al. The London Exercise And Pregnant smokers (LEAP) trial: a randomised controlled trial of physical activity for smoking cessation in pregnancy with an economic evaluation. Health Technol Assess 2015;19:vii-xxiv, 1–135.
	Barakat R, Ruiz JR, Stirling JR, Zakynthinaki M, Lucia A. Type of delivery is not affected by light resistance and toning exercise training during pregnancy: a randomized controlled trial. Am J Obstet Gynecol 2009;201:590.e1-6.
	Barakat R, Pelaez M, Lopez C, Montejo R, Coteron J. Exercise during pregnancy reduces the rate of cesarean and instrumental deliveries: results of a randomized controlled trial. J Matern Fetal Neonatal Med 2012;25:2372–6.
	Hart A, Morris N, Osborn SB, Wright HP. Effective uterine bloodflow during exercise in normal and pre-eclamptic pregnancies. Lancet. 1956;271:481–4.
	Barakat R, Pelaez M, Lopez C, Lucia A, Ruiz JR. Exercise during pregnancy and gestational diabetes-related adverse effects: a randomised controlled trial. Br J Sports Med 2013;47:630–6.
	Ferland S, Bujold E, Giguère Y, Girard M, Demers S, Forest JC. Association between physical activity in early pregnancy and markers of placental growth and function. J Obstet Gynaecol Can 2013;35:787–92.
**Definition of HDP not reported**	Garnaes KK, Morkved S, Salvesen O, Moholdt T. Exercise Training and Weight Gain in Obese Pregnant Women: A Randomized Controlled Trial (ETIP Trial). PLoS Med 2016;13:e1002079.
**CRF not reported in relation to HDP**	Korpi-Hyövälti E, Heinonen S, Schwab U, Laaksonen DE, Niskanen L. Effect of intensive counselling on physical activity in pregnant women at high risk for gestational diabetes mellitus. A clinical study in primary care. Prim Care Diabetes 2012;6:261–8.
	Avery MD, Leon AS, Kopher RA. Effects of a partially home-based exercise program for women with gestational diabetes. Obstet Gynecol 1997;89:10–5.
	Sagedal LR, Overby NC, Bere E, et al. Lifestyle intervention to limit gestational weight gain: the Norwegian Fit for Delivery randomised controlled trial. BJOG 2017;124:97–109.
	Hatch MC, Shu XO, McLean DE, et al. Maternal exercise during pregnancy, physical fitness, and fetal growth. Am J Epidemiol 1993;137:1105–14.
	Badon SE, Wander PL, Qiu C, Miller RS, Williams MA, Enquobahrie DA. Maternal Leisure Time Physical Activity and Infant Birth Size. Epidemiology 2016;27:74–81.
	Weissgerber TL, Davies GA, Roberts JM. Modification of angiogenic factors by regular and acute exercise during pregnancy. J Appl Physiol (1985) 2010;108:1217–23.
	Luoto R, Kinnunen TI, Aittasalo M, et al. Primary prevention of gestational diabetes mellitus and large-for-gestational-age newborns by lifestyle counseling: a cluster-randomized controlled trial. PLoS Med 2011;8:e1001036.
	Stafne SN, Salvesen KA, Romundstad PR, Eggebo TM, Carlsen SM, Morkved S. Regular exercise during pregnancy to prevent gestational diabetes: a randomized controlled trial. Obstet Gynecol 2012;119:29–36.
	Ruiz JR, Perales M, Pelaez M, Lopez C, Lucia A, Barakat R. Supervised exercise-based intervention to prevent excessive gestational weight gain: a randomized controlled trial. Mayo Clin Proc 2013;88:1388–97.
	Renault KM, Nørgaard K, Nilas L, et al. The Treatment of Obese Pregnant Women (TOP) study: a randomized controlled trial of the effect of physical activity intervention assessed by pedometer with or without dietary intervention in obese pregnant women. Am J Obstet Gynecol 2014;210:134.e1–9.
	Perales M, Santos-Lozano A, Sanchis-Gomar F, et al. Maternal Cardiac Adaptations to a Physical Exercise Program during Pregnancy. Med Sci Sports Exerc 2016;48:896–906.
	Bisson M, Croteau J, Guinhouya BC, et al. Physical activity during pregnancy and infant’s birth weight: results from the 3D Birth Cohort. BMJ Open Sport Exerc Med 2017;3:e000242.
**No CRF**	Abbasi R, Bakhshimoghaddam F, Alizadeh M. Major dietary patterns in relation to preeclampsia among Iranian pregnant women: a case-control study. J Matern Fetal Neonatal Med 2019:1–8.
	Aburezq M, AlAlban F, Alabdulrazzaq M, Badr H. Risk factors associated with gestational diabetes mellitus: The role of pregnancy-induced hypertension and physical inactivity. Pregnancy Hypertens 2020;22:64–70.
	Benschop L, Schelling SJ, Duvekot JJ, Roeters van Lennep JE. Cardiovascular health and vascular age after severe preeclampsia: A cohort study. Atherosclerosis 2020;292:136–42.
	Catov JM, Parker CB, Gibbs BB, et al. Patterns of leisure-time physical activity across pregnancy and adverse pregnancy outcomes. Int 2018;15:68.
	Chahal HS, Gelaye B, Mostofsky E, et al. Physical Exertion Immediately Prior to Placental Abruption: A Case-Crossover Study. Am J Epidemiol 2018;187:2073–9.
	Charkamyani F, Hosseinkhani A, Neisani Samani L, Khedmat L. Reducing the Adverse Maternal and Fetal Outcomes in IVF Women by Exercise Interventions During Pregnancy. Res Q Exerc Sport 2019;90:589–99.
	Do NC, Vestgaard M, Asbjornsdottir B, et al. Physical activity, sedentary behavior and development of preeclampsia in women with preexisting diabetes. Acta Diabetol 2020;57:559–67.
	Dodd JM, Deussen AR, Louise J. A Randomised Trial to Optimise Gestational Weight Gain and Improve Maternal and Infant Health Outcomes through Antenatal Dietary, Lifestyle and Exercise Advice: The OPTIMISE Randomised Trial. Nutrients 2019;11:02.
	Khoram S, Loripoor M, Pirhadi M, Beigi M. The effect of walking on pregnancy blood pressure disorders in women susceptible to pregnancy hypertension: A randomized clinical trial. J 2019;8:95.
	Lawan A, Apeyemi C, Chutiyami M, et al. Impact of physical activity and traumatic exposure on occurrence of gestational hypertension: a survey of pregnant women in an armed-conflict region in Nigeria. Hypertens 2020;39:295–301.
	Perales M, Valenzuela PL, Barakat R, et al. Gestational Exercise and Maternal and Child Health: Effects until Delivery and at Post-Natal Follow-up. J 2020;9:31.
	Raguema N, Benletaifa D, Mahjoub T, Lavoie JL. Increased physical activity is correlated with improved pregnancy outcomes in women with preeclampsia: A retrospective study. Pregnancy Hypertens 2020;21:118–23.
	To WW, Wong MW. Bone mineral density changes during pregnancy in actively exercising women as measured by quantitative ultrasound. Arch Gynecol Obstet 2012;286:357–63.
	Sehati Shafayi F, Akef M, Sadegi H, Sallakh Niknazhad A. A Comparison of Physical Activity and Nutritional Practices in Hypertensive and Non- hypertensive Pregnant Women. J 2012;4:53–6.
	Haelterman E, Marcoux S, Croteau A, Dramaix M. Population-based study on occupational risk factors for preeclampsia and gestational hypertension. Scandinavian Journal of Work, Environment and Health 2007;33:304–17.
	Rayl J, Holt VL, Williams MA, Nelson JL, Hickok DE, Weiss NS. A case-control study of preeclampsia and leisure-time physical activity. J Invest Med 1996;44.
	Bej P, Chhabra P, Sharma AK, Guleria K. Determination of Risk Factors for Pre-eclampsia and Eclampsia in a Tertiary Hospital of India: A Case Control Study. J 2013;2:371–5.
	Sorensen TK, Williams MA, Lee IM, Dashow EE, Thompson ML, Luthy DA. Recreational physical activity during pregnancy and risk of preeclampsia. Hypertension 2003;41:1273–80.
	Spinillo A, Capuzzo E, Colonna L, Piazzi G, Nicola S, Baltaro F. The effect of work activity in pregnancy on the risk of severe preeclampsia. Aust N Z J Obstet Gynaecol 1995;35:380–5.
	Marcoux S, Brisson J, Fabia J. The effect of leisure time physical activity on the risk of pre-eclampsia and gestational hypertension. J Epidemiol Community Health 1989;43:147–52.
	Fossum S, Halvorsen S, Vikanes AV, Roseboom TJ, Ariansen I, Naess O. Cardiovascular risk profile at the age of 40–45 in women with previous hyperemesis gravidarum or hypertensive disorders in pregnancy: A population-based study. Pregnancy Hypertens 2018;12:129–35.
	White E, Pivarnik J, Pfeiffer K. Resistance training during pregnancy and perinatal outcomes. J Phys Act Health 2014;11:1141–8.
	Magee RJ, Santillan MK, Betz AM, et al. Arterial stiffness but not physical activity levels and vascular endothelial function are altered in early/mid pregnancy in women who develop preeclampsia. FASEB Journal Conference: Experimental Biology 2018;32.
	Wergeland E, Strand K. Working conditions and prevalence of pre-eclampsia, Norway 1989. International Journal of Gynecology and Obstetrics 1997;58:189–96.
	Londero AP, Bertozzi S, Driul L, Marchesoni D. Couple risk factors for pregnancy: Related hypertensive disorders: A retrospective cohort study. Australian and New Zealand Journal of Obstetrics and Gynaecology 2009;49(5):570.
	Saurel-Cubizolles MJ, Kaminski M, Du Mazaubrun C, Breart G. Working conditions and arterial hypertension during pregnancy. [French]. Revue d’Epidemiologie et de Sante Publique 1991;39:37–43.
	Martin CL, Brunner Huber LR. Physical activity and hypertensive complications during pregnancy: findings from 2004 to 2006 North Carolina Pregnancy Risk Assessment Monitoring System. Birth 2010;37:202–10.
	Foo L, Mahendry A, McEniery C, Wilkinson I, Bennett P, Lees C. Pre-conception maternal haemodynamics is associated with subsequent development of preeclampsia (PE) or fetal growth restriction (FGR). BJOG: An International Journal of Obstetrics and Gynaecology 2018;125 (Supplement 1):12.
	Longo-Mbenza B, Tshimanga KB, Buassa-bu-Tsumbu B, Kabangu MJ. Diets rich in vegetables and physical activity are associated with a decreased risk of pregnancy induced hypertension among rural women from Kimpese, DR Congo. Niger J Med 2008;17:265–9.
	Liu J, Trivedi T, Blair SN, Ness A, Macdonald-Wallis C, Lawlor DA. Physical activity and hypertensive disorders of pregnancy among british women. Am J Epidemiol 2012;11:S22.
	Spracklen CN, Ryckman KK, Triche EW, Saftlas AF. Physical Activity During Pregnancy and Subsequent Risk of Preeclampsia and Gestational Hypertension: A Case Control Study. Matern Child Health J 2016;20:1193–202.
	Nelson N, Jenkins S, Lessard-Anderson C, Borowski K. Exercise habits during pregnancy and correlation with weight gain and obstetric outcomes. Obstetrics and Gynecology 2016;127 (Supplement 1):152S-3S.
	Clapp JF, 3rd. The effects of maternal exercise on early pregnancy outcome. Am J Obstet Gynecol 1989;161:1453–7.
	Rouleau CR, Tomfohr-Madsen LM, Campbell TS, Letourneau N, O’Beirne M, Giesbrecht GF. The role of maternal cardiac vagal control in the association between depressive symptoms and gestational hypertension. Biological Psychology 2016;117:32–42.
	Timpka S, Stuart JJ, Tanz LJ, Rimm EB, Franks PW, Rich-Edwards JW. Lifestyle in progression from hypertensive disorders of pregnancy to chronic hypertension in Nurses’ Health Study II: observational cohort study. Bmj 2017;358:j3024.
	Vladutiu CJ, Evenson KR, Jukic AM, Herring AH. Correlates of Self-Reported Physical Activity at 3 and 12 Months Postpartum. J Phys Act Health 2015;12:814–22.
	Egeland GM, Klungsoyr K, Oyen N, Tell GS, Naess O, Skjaerven R. Preconception Cardiovascular Risk Factor Differences between Gestational Hypertension and Preeclampsia: Cohort Norway Study. Hypertension 2016;67:1173–80.
	Chasan-Taber L, Silveira M, Pekow P, et al. Physical activity, sedentary behavior and risk of hypertensive disorders of pregnancy in Hispanic women. Hypertens 2015;34:1–16.
	Ng SK, Cameron CM, Hills AP, McClure RJ, Scuffham PA. Socioeconomic disparities in prepregnancy BMI and impact on maternal and neonatal outcomes and postpartum weight retention: the EFHL longitudinal birth cohort study. BMC Pregnancy Childbirth 2014;14:314.
	Currie LM, Woolcott CG, Fell DB, Armson BA, Dodds L. The association between physical activity and maternal and neonatal outcomes: a prospective cohort. Matern Child Health J 2014;18:1823–30.
	Fortner RT, Pekow PS, Whitcomb BW, Sievert LL, Markenson G, Chasan-Taber L. Physical activity and hypertensive disorders of pregnancy among Hispanic women. Med Sci Sports Exerc 2011;43:639–46.
	Vollebregt KC, Wolf H, Boer K, van der Wal MF, Vrijkotte TG, Bonsel GJ. Does physical activity in leisure time early in pregnancy reduce the incidence of preeclampsia or gestational hypertension? Acta Obstet Gynecol Scand 2010;89:261–7.
	Osterdal ML, Strom M, Klemmensen AK, et al. Does leisure time physical activity in early pregnancy protect against pre-eclampsia? Prospective cohort in Danish women. Bjog 2009;116:98–107.
	Rudra CB, Sorensen TK, Luthy DA, Williams MA. A prospective analysis of recreational physical activity and preeclampsia risk. Med Sci Sports Exerc 2008;40:1581–8.
	Saftlas AF, Logsden-Sackett N, Wang W, Woolson R, Bracken MB. Work, leisure-time physical activity, and risk of preeclampsia and gestational hypertension. Am J Epidemiol 2004;160:758–65.
	Catov JM, Parker CB, Gibbs BB, Bann C, Carper B, Grobman WA. Patterns of physical activity across pregnancy and their association with adverse pregnancy outcomes. Circulation Conference: American Heart Association’s Epidemiology and Prevention/Lifestyle and Cardiometabolic Health 2017;135.
	Mutsaerts MAQ, Groen H, Buiter-Van Der Meer A, et al. Effects of paternal and maternal lifestyle factors on pregnancy complications and perinatal outcome. A population-based birth-cohort study: The GECKO Drenthe cohort. Hum Reprod 2014;29:824–34.
	Tyldum EV, Romundstad PR, Slordahl SA. Pre-pregnancy physical activity and preeclampsia risk: a prospective population-based cohort study. Acta Obstet Gynecol Scand 2010;89:315–20.
	Magnus P, Trogstad L, Owe KM, Olsen SF, Nystad W. Recreational physical activity and the risk of preeclampsia: a prospective cohort of Norwegian women. Am J Epidemiol 2008;168:952–7.
	Hegaard HK, Ottesen B, Hedegaard M, et al. The association between leisure time physical activity in the year before pregnancy and pre-eclampsia. J Obstet Gynaecol 2010;30:21–4.
	Claesson R, Arehall A, Strevens H, Wide-Swensson D. Markers of future cardiovascular disease in women with previous hypertension in pregnancy. International Journal of Gynecology and Obstetrics 2009;2:S144.
	Phan K, Bidulka P, Gomez YH, et al. Effect of Acute Weight Gain and Physical Activity on Arterial Stiffness in Pregnant Women at High Risk for Hypertensive Disorders of Pregnancy. Can J Cardiol 2016;32:S4-S5.
	Petrella E, Malavolti M, Bertarini V, et al. Gestational weight gain in overweight and obese women enrolled in a healthy lifestyle and eating habits program. J Matern Fetal Neonatal Med 2014;27:1348–52.
	Poston L. Obesity in pregnancy; the role of nutrition in the health of mother and child. Annals of Nutrition and Metabolism 2015;1:26.
	Hoirisch-Clapauch S, Constan Werneck Sant’Anna M, Cinelli Couto Moreira E, et al. Lifestyle modification increases the take-home baby rate in women with recurrent early miscarriages: A randomised study. Thrombosis Research 2017;151 (Supplement 1):S104.
	da Silva SG, Hallal PC, Domingues MR, et al. A randomized controlled trial of exercise during pregnancy on maternal and neonatal outcomes: results from the PAMELA study. Int 2017;14:175.
	Wang C, Wei Y, Zhang X, et al. A randomized clinical trial of exercise during pregnancy to prevent gestational diabetes mellitus and improve pregnancy outcome in overweight and obese pregnant women. Am J Obstet Gynecol 2017;216:340–51.
	Barakat R, Pelaez M, Cordero Y, et al. Exercise during pregnancy protects against hypertension and macrosomia: randomized clinical trial. Am J Obstet Gynecol 2016;214:649.e1–8.
	Aardenburg R, Spaanderman ME, van Eijndhoven HW, de Leeuw PW, Peeters LL. Formerly preeclamptic women with a subnormal plasma volume are unable to maintain a rise in stroke volume during moderate exercise. J Soc Gynecol Investig 2005;12:599–603.
	Tomic V, Sporis G, Tomic J, Milanovic Z, Zigmundovac-Klaic D, Pantelic S. The effect of maternal exercise during pregnancy on abnormal fetal growth. Croatian Medical Journal 2013;54:362–8.
	Rakhshani A, Nagarathna R, Mhaskar R, Mhaskar A, Thomas A, Gunasheela S. The effects of yoga in prevention of pregnancy complications in high-risk pregnancies: A randomized controlled trial. Prev Med 2012;55:333–40.
	Vinter C, Jensen DM, Beck-Nielsen H, Jorgensen JS, Ovesen PG. The clinical effect of lifestyle intervention during pregnancy and obese women. Acta Obstet Gynecol Scand 2012;159:54–5.
	Vinter CA, Jensen DM, Ovesen PG, Beck-Nielsen H, J.S JO. Lifestyle and pregnancy (LIP) study: The clinical effect of lifestyle intervention during pregnancy in obese women. Diabetes 2011;1:A348-A9.
	Kasawara KT, Burgos CSG, Do Nascimento SL, Ferreira NO, Surita FG, Pinto ESJL. Maternal and perinatal outcomes of exercise in pregnant women with chronic hypertension and/or previous preeclampsia: A randomized controlled trial. ISRN Obstetrics and Gynecology 2013;2013 (no pagination).
	Lombardi W, Wilson S, Peniston PB. Wellness intervention with pregnant soldiers. Mil Med 1999;164:22–9.
	Kvehaugen AS, Andersen LF, Staff AC. Dietary intake and physical activity in women and offspring after pregnancies complicated by preeclampsia or diabetes mellitus. Acta Obstet Gynecol Scand 2010;89:1486–90.
	Rees GB, Broughton Pipkin F, Symonds EM, Patrick JM. A longitudinal study of respiratory changes in normal human pregnancy with cross-sectional data on subjects with pregnancy-induced hypertension. Am J Obstet Gynecol 1990;162:826–30.
	Barakat R, Perales M, Bacchi M, Coteron J, Refoyo I. A program of exercise throughout pregnancy. Is it safe to mother and newborn? Am J Health Promot 2014;29:2–8.
	Adank MC, Broere-Brown ZA, Goncalves R, et al. Maternal cardiovascular adaptation to twin pregnancy: a population-based prospective cohort study. BMC Pregnancy Childbirth 2020;20:327.
	Cordero Rodríguez Y, Peláez Puente M, Abad M, Perales Santaella M, Barakat Carballo R. [Can moderate physical exercise during pregnancy act as a factor in preventing Gestational Diabetes?] International Journal of Sport Science 2012;27(8):3–19.
	Elden H, Ostgaard HC, Fagevik-Olsen M, Ladfors L, Hagberg H. Treatments of pelvic girdle pain in pregnant women: adverse effects of standard treatment, acupuncture and stabilising exercises on the pregnancy, mother, delivery and the fetus/neonate. BMC Complement Altern Med 2008;8:34.
	Kong KL, Campbell CG, Foster RC, Peterson AD, Lanningham-Foster L. A pilot walking program promotes moderate-intensity physical activity during pregnancy. Med Sci Sports Exerc 2014;46:462–71.
	de Oliveria Melo AS, Silva JL, Tavares JS, Barros VO, Leite DF, Amorim MM. Effect of a physical exercise program during pregnancy on uteroplacental and fetal blood flow and fetal growth: a randomized controlled trial. Obstet Gynecol 2012;120:302–10.
	Narendran S, Nagarathna R, Narendran V, Gunasheela S, Nagendra HR. Efficacy of yoga on pregnancy outcome. J Altern Complement Med 2005;11:237–44.
	Harris ST, Liu J, Wilcox S, Moran R, Gallagher A. Exercise during pregnancy and its association with gestational weight gain. Matern Child Health J 2015;19:528–37.
	Dale E, Mullinax KM, Bryan DH. Exercise during pregnancy: effects on the fetus. Can J Appl Sport Sci 1982;7:98–103.
	Koivusalo SB, Rönö K, Klemetti MM, et al. Gestational Diabetes Mellitus Can Be Prevented by Lifestyle Intervention: The Finnish Gestational Diabetes Prevention Study (RADIEL): A Randomized Controlled Trial. Diabetes Care 2016;39:24–30.
	Taniguchi C, Sato C. Home-based walking during pregnancy affects mood and birth outcomes among sedentary women: A randomized controlled trial. Int J Nurs Pract 2016;22:420–6.
	Magann EF, Evans SF, Weitz B, Newnham J. Antepartum, intrapartum, and neonatal significance of exercise on healthy low-risk pregnant working women. Obstet Gynecol 2002;99:466–72.
	Downs DS, Hausenblas HA. Pregnant women’s third trimester exercise behaviors, body mass index, and pregnancy outcomes. Psychology and Health 2007;22:545–559.
	Snapp CA, Donaldson SK. Gestational diabetes mellitus: physical exercise and health outcomes. Biol Res Nurs 2008;10:145–55.
	Seneviratne SN, Jiang Y, Derraik J, et al. Effects of antenatal exercise in overweight and obese pregnant women on maternal and perinatal outcomes: a randomised controlled trial. BJOG 2016;123:588–97.
	Putnam KF, Mueller LA, Magann EF, et al. Evaluating effects of self-reported domestic physical activity on pregnancy and neonatal outcomes in “stay at home” military wives. Mil Med 2013;178:893–8.
	Hollingsworth DR, Moore TR. Postprandial walking exercise in pregnant insulin-dependent (type I) diabetic women: reduction of plasma lipid levels but absence of a significant effect on glycemic control. Am J Obstet Gynecol 1987;157:1359–63.
	Misiani R, Marchesi D, Tiraboschi G, et al. Urinary albumin excretion in normal pregnancy and pregnancy-induced hypertension. Nephron 1991;59:416–22.
	Abdulaziz KS, Draz AH. Reflexology versus traditional physical therapy program in pre-eclampsic pregnant women with ankle oedema. International Journal of PharmTech Research 2016;9:8–15.
	Dennis AT, Hardy L, Leeton L. The prone position in preeclampsia. International Journal of Obstetric Anesthesia 2016;1:S32.
	Drost JT, Arpaci G, Van Eyck J, Van Der Schouw YT, Maas AHEM. Prevalence and predictors of the metabolic syndrome in women 10 years post preeclampsia. Data from the Preeclampsia Risk EValuation in FEMales (PREVFEM) study. Eur Heart J 2010;1:1007.
	Kim C, Brawarsky P, Jackson RA, Fuentes-Afflick E, Haas JS. Changes in health status experienced by women with gestational diabetes and pregnancy-induced hypertensive disorders. J Womens Health (Larchmt) 2005;14:729–36.
	Agatisa PK, Ness RB, Roberts JM, Costantino JP, Kuller LH, McLaughlin MK. Impairment of endothelial function in women with a history of preeclampsia: an indicator of cardiovascular risk. Am J Physiol Heart Circ Physiol 2004;286:H1389–93.
	Kaplan R, Jay M. The impact of a behavioral intervention on weight gain during pregnancy. Journal of Clinical Outcomes Management 2011;18:449–51.
	Hogstedt S, Rane A. Plasma concentration–Effect relationship of metoprolol during and after pregnancy. European Journal of Clinical Pharmacology 1993;44:243–6.
	Uma R, Bhavadharini B, Ranjani H, et al. Pregnancy outcome of gestational diabetes mellitus using a structured model of care: WINGS project (WINGS-10). J Obstet Gynaecol Res 2017;43:468–75.
	Bobic MV, Habek D, Habek JC. Perinatal epidemiological risk factors for preeclampsia. Acta Clin 2015;54:9–13.
	Caughey MC, Cho JS, Wu YK, Nix WB, Yeo S. Feasibility of stretching exercise for prevention of late-onset preeclampsia: A pilot trial. Circulation Conference: American Heart Association’s Epidemiology and Prevention/Lifestyle and Cardiometabolic Health 2018;137.
	Savitsky LM, Valent A, Burwick R, Marshall N, Caughey AB. Cost-effectiveness of exercise for the prevention of preeclampsia and gestational diabetes in normal weight women. American Journal of Obstetrics and Gynecology 2017;216 (1 Supplement 1):S486.
	Cordero Y, Mottola MF, Vargas J, Blanco M, Barakat R. Exercise Is Associated with a Reduction in Gestational Diabetes Mellitus. Med Sci Sports Exerc 2015;47:1328–33.
**Duplicate population**	Barakat R, Pelaez M, Montejo R, Refoyo I, Coteron J. Exercise throughout pregnancy does not cause preterm delivery: a randomized, controlled trial. J Phys Act Health 2014;11:1012–7.
	Rauramo I. Effect of short-term physical exercise on foetal heart rate and uterine activity in normal and abnormal pregnancies. Ann Chir Gynaecol 1987;76:274–9.
	Rudra CB, Williams MA, Lee IM, Miller RS, Sorensen TK. Perceived exertion during prepregnancy physical activity and preeclampsia risk. Med Sci Sports Exerc 2005;37:1836–41.
	Scholten RR, Spaanderman MEA, Green DJ, Hopman MTE, Thijssen DHJ. Retrograde shear rate in formerly preeclamptic and healthy women before and after exercise training: Relationship with endothelial function. American Journal of Physiology–Heart and Circulatory Physiology 2014;307:H418-H25.
	Scholten RR, Thijssen D, Lotgering FK, Hopman MTE, Spaanderman MEA. Vascular adaptations to 12-weeks cycling training in formerly preeclamptic women. Reprod Sci 2012;1:220A.
	Scholten RR, Thijssen DJ, Lotgering FK, Hopman MT, Spaanderman ME. Cardiovascular effects of aerobic exercise training in formerly preeclamptic women and healthy parous control subjects. Am J Obstet Gynecol 2014;211:516.e1-.e11.
	Harrington BC. Response to exercise in early pregnancy may predict preeclampsia. Am Fam Physician 1996;54:2523–4.
	Petrella E, Bruno R, Pedrielli G, Bertarini V, Neri I, Facchinetti F. An early-customized low glycaemic-index (GI) diet prevents both the gestational diabetes mellitus (GDM) and the large for gestational age (LGA) babies occurrence in overweight/obese pregnant women. Gynecological Endocrinology 2016;32 (Supplement 1):71.
	Petrella E, Tamborrino V, Bertarini V, Neri I, Facchinetti F. An early-customized low glycaemic-index (GI) diet prevents adverse pregnancy outcomes in overweight/obese women. Journal of Maternal-Fetal and Neonatal Medicine 2016;29 (Supplement 1):94–5.
	Egeland G, Klungsoyr K, Ebbing M, et al. Preconception cardiovascular determinants of gestational hypertension: Cohort Norway and the Medical Birth Registry of Norway. European Journal of Epidemiology 2013;1:S23.
	Ignatko Irina V. Psychosomatic status of pregnant women with arterial hypertension and obesity. J Perinat Med 2017;45 (Supplement 2):337.
	Morris EA, McBride CA, Badger GJ, Bernstein IM. Low prepregnancy plasma volume and increased blood pressure are associated with increased risk of small for gestational age infants in subsequent pregnancy. Reprod Sci 2016;1:138A-9A.
	Morris E, McBride C, Badger G, Bernstein I. Women with a history of HELLP syndrome display different cardiovascular features than women with a history of preeclampsia. American Journal of Obstetrics and Gynecology 2015;1:S169.
	Bahri Khomami M, Moran L, Grieger J, et al. Pregnancy complications in women with and without PCOS following consideration of modifiable lifestyle factors: The scope cohort study. Australian and New Zealand Journal of Obstetrics and Gynaecology 2017;57 (Supplement 1):34.
	Petrella E, Facchinetti F, Bertarini V, Pignatti L, Neri I, Battistini NC. Occurrence of pregnancy complications in women with BMI >25 submitted to a healthy lifestyle and eating habits program. American Journal of Obstetrics and Gynecology 2013;208 (1 SUPPL.1):S33-S4.
	Yeo S, Steele NM, Chang MC, Leclaire SM, Ronis DL, Hayashi R. Effect of exercise on blood pressure in pregnant women with a high risk of gestational hypertensive disorders. J Reprod Med 2000;45:293–8.
	Kasawara KT, Burgos CS, Costa ML, JL ES. PP046. Adherence to exercise with bicycle during pregnancy in women with risk of preeclampsia. Pregnancy Hypertens 2012;2:266–7.
	Kasawara KT, Burgos CS, Nascimento SL, Costa ML, Surita F, JL ES. OS020. Effects of exercise on maternal and neonatal outcomes in pregnantwomen with chronic hypertension and/or previous preecampsia: A randomized clinical trial. Pregnancy Hypertens 2012;2:185–6.
	Kasawara KT, Burgos CSG, Pinto ESJLDC. Assessment of physical exercise for pregnant women with risk for preeclampsia development: Preliminary data. Journal of Perinatal Medicine Conference: 10th World Congress of Perinatal Medicine 2011;39.
	Burgos CS, Kasawara KT, Costa ML, Pinto ESJL. PP041. The effect of exercise in pregnant women with chronic hypertension and/or previous preeclampsia on blood pressure and heart rate variability. Pregnancy Hypertens 2012;2:263–4.
	Rouleau CR, Tomfohr LM, Campbell TS, Letourneau N, Giesbrecht GF. Lower high-frequency heart rate variability in early pregnancy mediates the association between maternal depressed mood and incident gestational hypertension. Psychosomatic Medicine 2015;77(3):A133.
	Catov J, Parker C, Gibbs B, Carper B, Grobman W. Patterns of physical activity from early pregnancy through five years after delivery and their association with maternal cardiometabolic health. American journal of obstetrics and gynecology Conference: 37th annual meeting of the society for maternal-fetal medicine: the pregnancy meeting United states Conference start: 20170123 Conference end: 201701282017:S50.
	Berks D, Hoedjes M, Raat H, Duvekot HJ, Steegers EAP. Effects of lifestyle intervention after complicated pregnancy: Results of the Pro-Active study. Pregnancy Hypertens 2015;5(1):36–7.
	Mutsaerts MAQ, Groen H, Buiter-Van Der Meer A, et al. Effects of paternal and maternal lifestyle factors on pregnancy complications and perinatal outcome. A Dutch population-based birth-cohort study: The GECKO Drenthe study. Human Reproduction Conference: 28th Annual Meeting of the European Society of Human Reproduction and Embryology, ESHRE 2012;27.
	Berks D, Hoedjes M, Franx A, Duvekot HJ, Raat H, Steegers EA. Lifestyle intervention after complicated pregnancy successfully improves cardiovascular and metabolic health: Results of the pro-active study. Pregnancy Hypertens 2012;2(3):192–3.
	Kasawara KT, Burgos CSG, Nascimento SL, Costa ML, Surita F, Pinto ESJL. Effects of exercise on maternal and neonatal outcomes in pregnant women with chronic hypertension and/or previous preecampsia: A randomized clinical trial. Pregnancy Hypertens 2012;2(3):185–6.
	Currie L, Woolcott C, Fell DF, Armson BA, Dodds L. Physical activity and pregnancy outcomes. Am J Epidemiol 2011;11:S214.
	Yeo S. Prenatal stretching exercise and autonomic responses: preliminary data and a model for reducing preeclampsia. J Nurs Scholarsh 2010;42:113–21.
	Berks D, Hoedjes M, Raat H, Franx A, Duvekot HJ, Steegers EA. OS109. Lifestyle intervention after complicated pregnancy successfully improves saturated fat-intake, but not exercise and smoking habits: results of the pro-active study. Pregnancy Hypertens 2012;2:239.
	Timpka S, Stuart JJ, Tanz LJ, Rimm EB, Franks PW, Rich-Edwards JW. Progression from hypertensive disorders of pregnancy to chronic hypertension: Obesity appears especially detrimental. Circulation Conference: American Heart Association’s 2016;134.
	McBride CA, Morris EA, Badger GJ, Bernstein IM. Physical fitness and cardiovascular phenotype in young women. Reprod Sci 2015;1:203A.
