# Supplementary material for: Association between Cardiorespiratory Fitness and Hypertensive Disorders of Pregnancy: A Systematic Review and Meta-Analysis
Source: J Clin Med. 2022 Jul 27;11(15):4364. doi: 10.3390/jcm11154364 (PMC9369055; doi:10.3390/jcm11154364)
Supplement: Supplementary file 1 [file jcm-11-04364-s001.zip › jcm-1791538-supplementary.pdf]

(a)

Study or Subgroup	During HDP pregnancy Mean	SD	Total	During no HDP pregnancy Mean	SD	Total	Weight	Mean Difference IV, Random, 95% CI
Bisson 2014	2.3	0.4	3	1.9	0.3	56	12.1%	0.40 [-0.06, 0.86]
Yeo 2008	1.6914	0.4572	35	1.53	0.33226495	69	87.9%	0.16 [-0.01, 0.33]
**Total (95% CI)**			**38**			**125**	**100.0%**	**0.19 [0.03, 0.35]**

Heterogeneity: Tau² = 0.00; Chi² = 0.91, df = 1 (P = 0.34); I² = 0%
Test for overall effect: Z = 2.33 (P = 0.02)

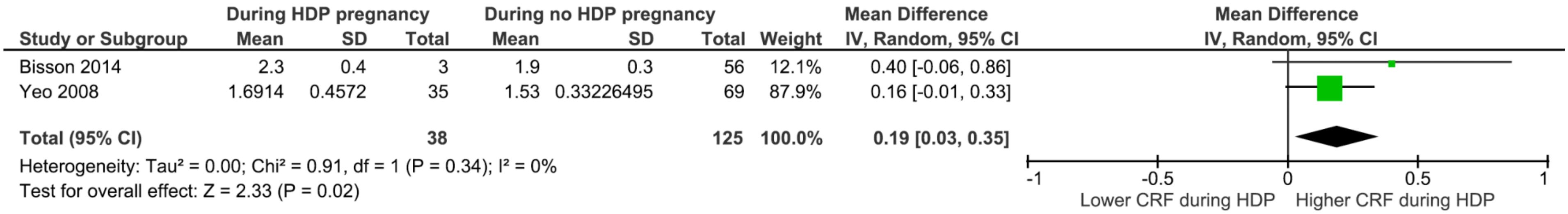

(b)

Study or Subgroup	Developed PE Mean	SD	Total	Did not develop PE Mean	SD	Total	Weight	Std. Mean Difference IV, Random, 95% CI
Bisson 2014	2.6	0.2	2	1.9	0.3	56	27.0%	2.31 [0.84, 3.79]
Guelfi 2016	1.74	0.18	3	1.52	0.25	137	32.4%	0.88 [-0.27, 2.03]
Yeo 2008	1.57	0.31622777	10	1.53	0.33226495	69	40.6%	0.12 [-0.54, 0.78]
**Total (95% CI)**			**15**			**262**	**100.0%**	**0.96 [-0.23, 2.14]**

Heterogeneity: Tau² = 0.79; Chi² = 7.40, df = 2 (P = 0.02); I² = 73%
Test for overall effect: Z = 1.58 (P = 0.11)

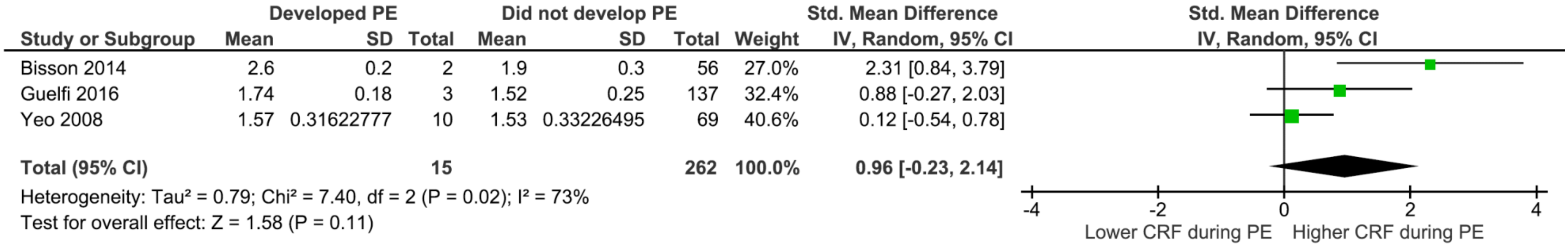

(c)

Study or Subgroup	During GH pregnancy Mean	SD	Total	During no GH pregnancy Mean	SD	Total	Std. Mean Difference IV, Random, 95% CI
Yeo 2008	1.74	0.5	25	1.53	0.33226495	69	0.54 [0.08, 1.01]

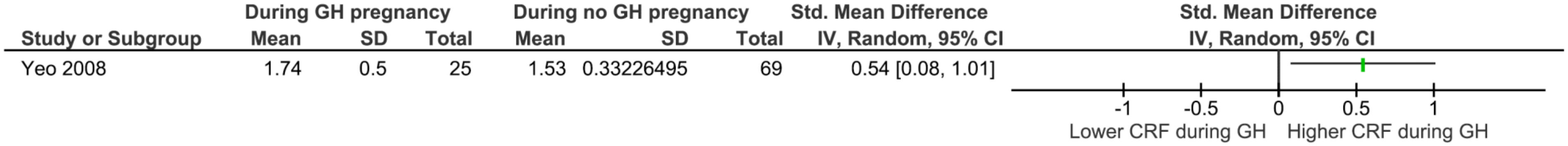

Figure S1. Mean weight-unadjusted differences in VO2max during pregnancy and subsequent development of preeclampsia and/or gestational hypertension: (**a**) Weight-unadjusted VO2max (in L/min) and preeclampsia or gestational hypertension; (**b**) Weight-unadjusted VO2max (in L/min) and preeclampsia; (**c**) Weight-unadjusted VO2max (in L/min) and gestational hypertension.

Study or Subgroup	Developed HDP Mean	Developed HDP SD	Developed HDP Total	Did not develop HDP Mean	Did not develop HDP SD	Did not develop HDP Total	Weight	Std. Mean Difference IV, Random, 95% CI
Price 2012	957	308	3	1,089	190	59	39.5%	-0.67 [-1.83, 0.50]
Rauramo 1988	91	18	13	85	20	13	60.5%	0.31 [-0.47, 1.08]
**Total (95% CI)**			**16**			**72**	**100.0%**	**-0.08 [-1.01, 0.85]**

Heterogeneity: $Tau^2 = 0.22$; $Chi^2 = 1.86$, df = 1 (P = 0.17); $I^2 = 46\%$

Test for overall effect: Z = 0.17 (P = 0.87)

Std. Mean Difference IV, Random, 95% CI

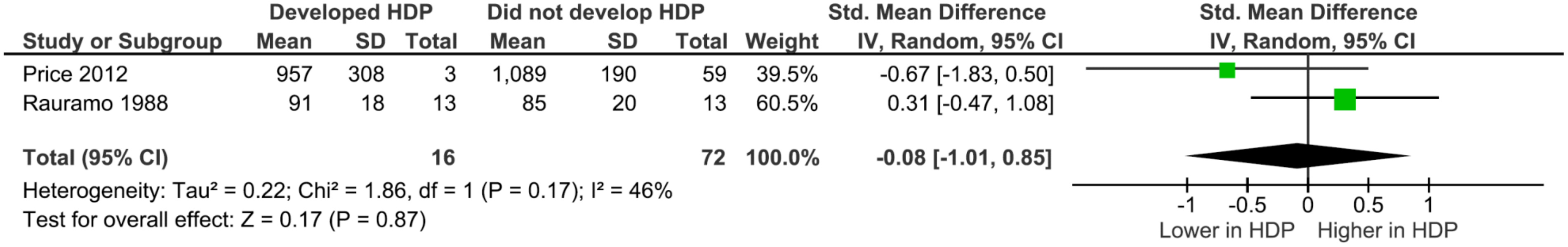

Figure S2. Mean difference in work rate (Watts) during pregnancy and development of preeclampsia and/or gestational hypertension.
